# Supplementary material for: The effect of maternal care on gene expression and DNA methylation in a subsocial bee
Source: Nat Commun. 2018 Aug 27;9:3468. doi: 10.1038/s41467-018-05903-0 (PMC6110825; doi:10.1038/s41467-018-05903-0)
Supplement: Supplementary file 1 — Supplementary Information [file 41467_2018_5903_MOESM1_ESM.pdf]

Supplementary Information

The effect of maternal care on gene expression and DNA methylation in a subsocial bee

Arsenault et al.

Supplementary Figures

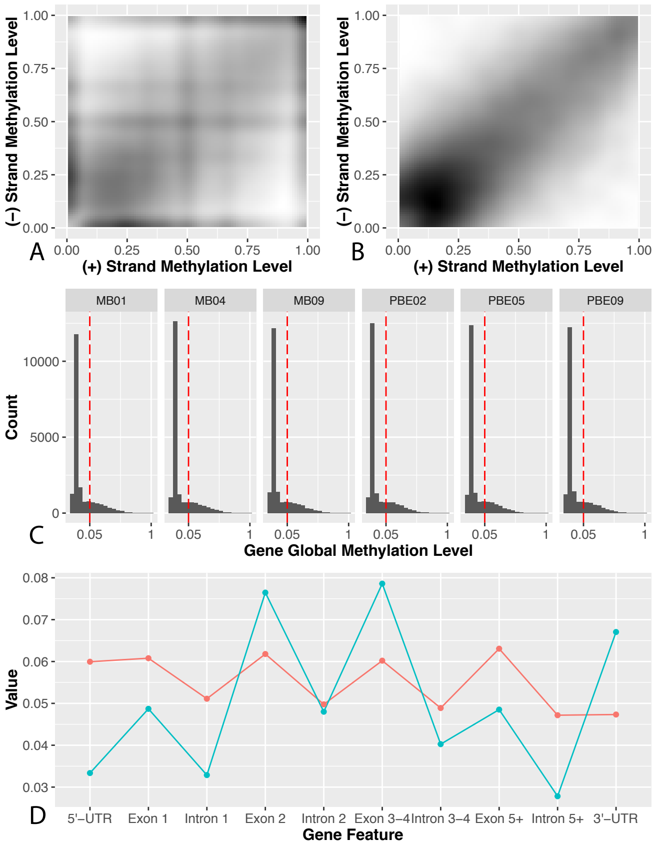

Supplementary Figure 1: General Methylation analysis. (A) Symmetry of CpG dinucleotides when no coverage threshold is imposed. (B) Symmetry of CpG dinucleotides when a coverage threshold of 10 (per Cytosine) is imposed. (C) Histogram of methylation levels in genes. Red line shows the threshold used to define methylated genes. (D) CpG Density (Red) and Methylation Level (Blue) by gene feature. With these analyses, we find expected genomic targets of DNA methylation and justify merging strands for analysis of DNA methylation at CpGs.

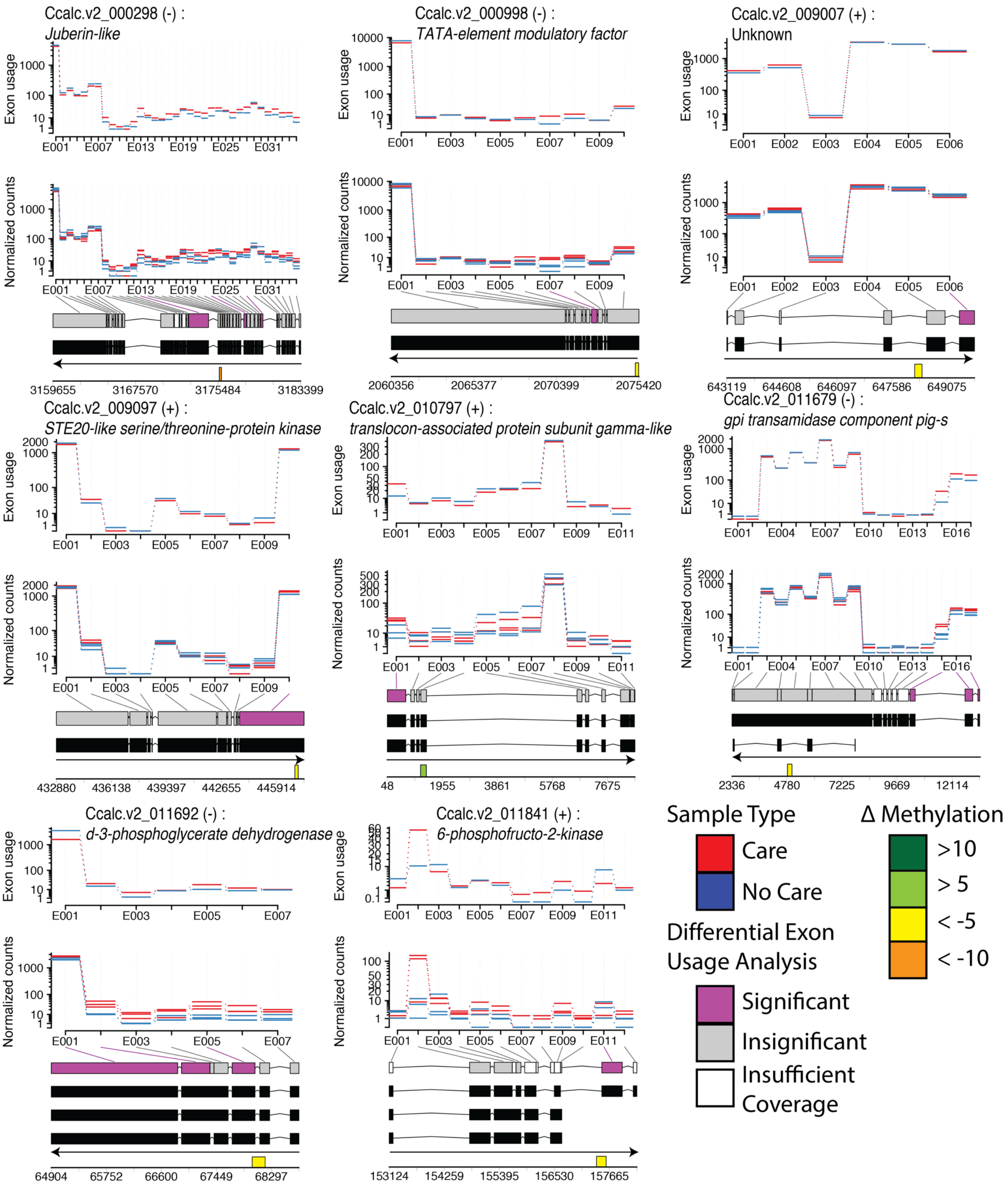

Supplementary Figure 2: Minimal and random overlap between differential methylation and differential exon usage. 8 genes that contain significantly differentially utilized exons also contained DMRs. Of these 8 genes, 3 of them showed the DMR overlapping with a differentially utilized exon (Ccalc.v2_009097, Ccalc.v2_011692, and Ccalc.v2_011841). The top panel shows average exon usage for each treatment group. Beneath this are the counts for each exon in each sample normalized based on overall expression of the given gene. The gene model is represented below each exon usage plot with purple exons indicating statistical significance (FDR-corrected p-value < 0.01). Transcription direction is indicated by an arrow and DMR's are overlaid beneath the gene model with colors indicating the change in methylation level.

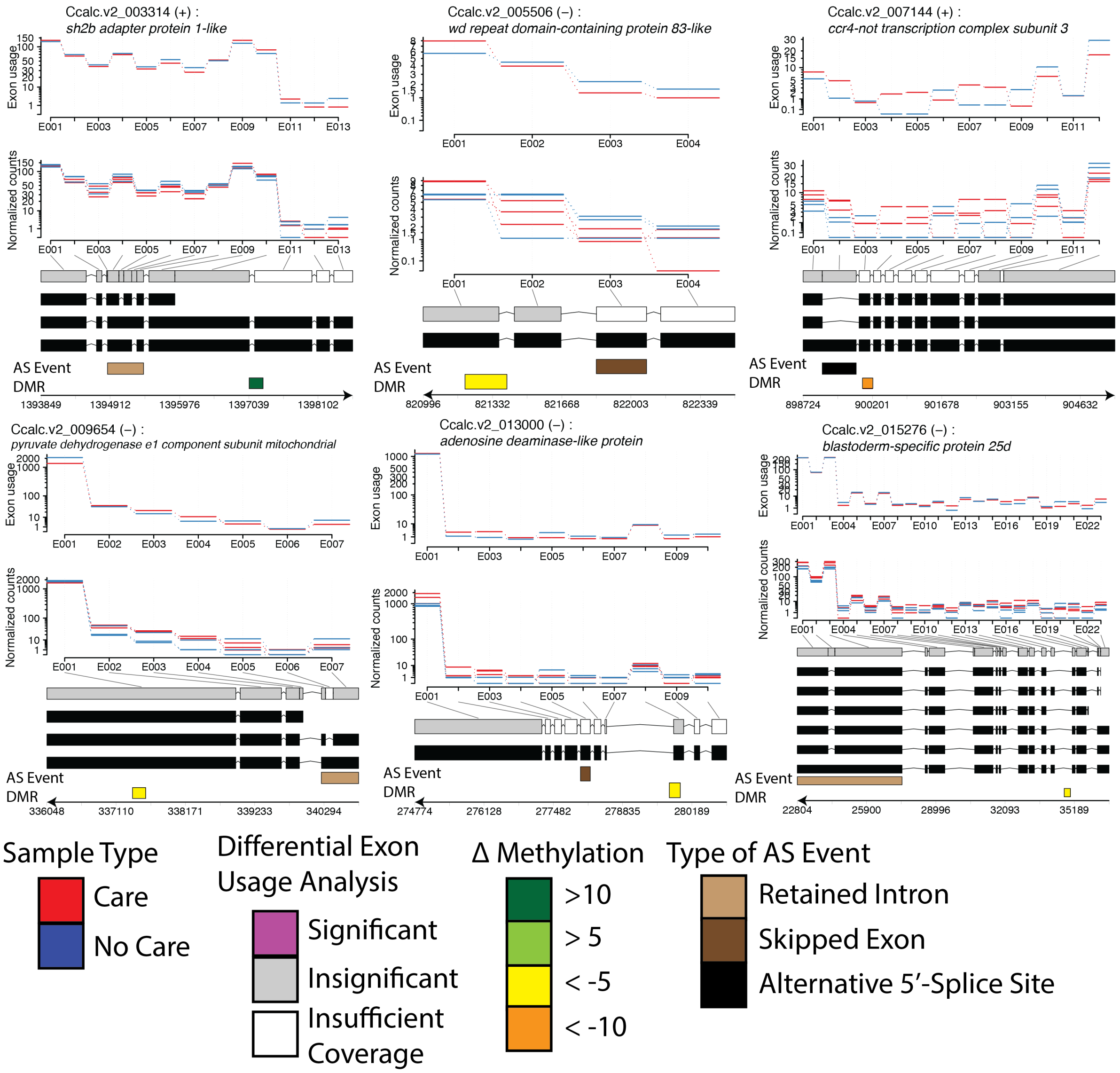

Supplementary Figure 3: Minimal and random overlap between differential methylation and rMATS alternative splicing. 8 genes that contain significantly differentially utilized exons also contained DMRs. Of these 8 genes, 3 of them showed the DMR overlapping with a differentially utilized exon (Ccalc.v2_009097, Ccalc.v2_011692, and Ccalc.v2_011841). The top panel shows average exon usage for each treatment group. Beneath this are the counts for each exon in each sample normalized based on overall expression of the given gene. The gene model is represented below each exon usage plot with purple exons indicating statistical significance (FDR-corrected p-value < 0.01). Significant alternative splicing events (rMATS, FDR-corrected p-value < 0.01) are marked below the isoforms and color coded based on their type. Transcription direction is indicated by an arrow and DMR's are overlaid beneath the gene model with colors indicating the change in methylation level.

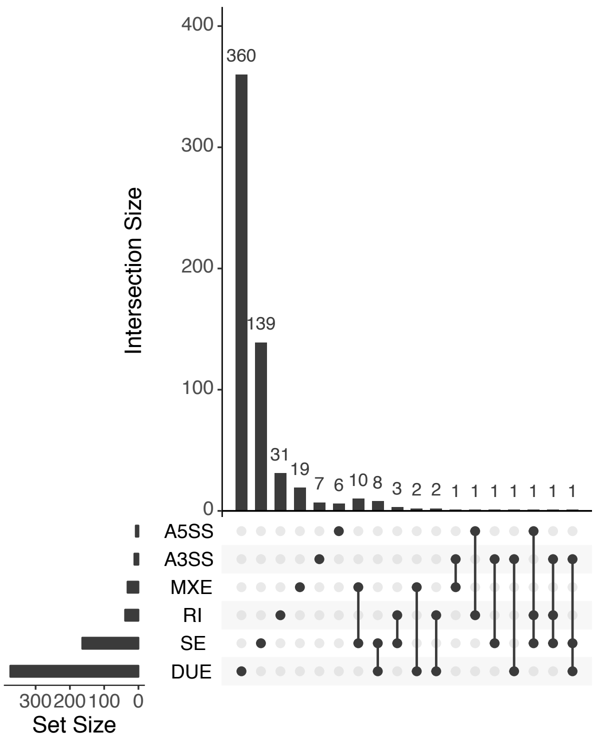

Supplementary Figure 4: DEXseq and rMATS alternative splicing methods yield non-redundant alternatively spliced genes. UpSet plot illustrating the intersection between different types of alternatively spliced genes computed using rMATS and DEXseq. The nature of a given intersection is indicated by the dots below the bar plot. Genes with alternative 5' splice sites are categorized as A5SS. Genes with alternative 3' splice sites are categorized as A3SS. Genes with mutually exclusive exons are categorized as MXE. Genes with retained introns are categorized as RI. Genes skipped exons are categorized as SE. All of the above categories were computed using rMATS. Genes with differentially utilized exons, computed by DEXseq, are categorized as DUE. All genes were considered alternatively spliced given an FDR-corrected p-value < 0.01 in the programs' respective methods.

Supplementary Tables

Supplementary Table 1: No association was detected between genes under positive selection and genes that are differential expressed (DEGs) in response to the loss of maternal care.

	Positively Selected Genes	Not Positively Selected
DEGs	72<br>(69.4)	642<br>(644.6)
Not DEGs	421<br>(423.6)	3937<br>(3934.4)

Chi-square = 0.1255, p = 0.723

Supplementary Table 2: No association was detected between genes under positive selection and genes that contain differentially utilized(DU) exons (A) or any other detected form of alternative splicing(AS)(B) in response to the loss of maternal care.

(A)	Contains DU Exons	Does not contain DU Exons	(B)	Contains DU Exon or AS Event	Does not contains DU Exon or AS Event
Positively Selected Genes	20<br>(19.9)	488<br>(488.1)		32<br>(34.4)	476<br>(473.6)
Not Positively Selected	182<br>(182.1)	4458<br>(4459.9)		317<br>(314.6)	4329<br>(4331.4)
	Chi-square = 0.00026	p = 0.99		Chi-square = 0.199	p = 0.66

Supplementary Table 3: A strong negative association was detected between genes under positive selection and genes that contain greater than 5% methylation in at least one of our six samples.

	Positively Selected Genes	Not Positively Selected
Methylated Genes	194<br>(250.4)	2410<br>(2353.6)
Unmethylated Genes	335<br>(278.6)	2563<br>(2619.4)

Chi-square = 26.66, p = 2.43e-07

Supplementary Table 4: No association was detected between genes under positive selection and genes that contain differentially methylated regions(DMR) with respect to the loss of maternal care.

	Positively Selected Genes	Not Positively Selected
Contains DMR	18<br>(15.7)	141<br>(143.3)
Does Not Contain DMR	325<br>(327.3)	2985<br>(2982.7)

Chi-square = 0.3842, p = 0.535

Supplementary Table 5: No association was detected between genes containing differentially methylated regions (DMRs) and genes that are differential expressed (DEGs) in response to the loss of maternal care.

	Genes containing DMR	Genes without DMR
DEGs	14<br>(18.0)	385<br>(381.0)
Not DEGs	202<br>(198.0)	4179<br>(4183.0)

Chi-square = 1.0294, p = 0.310

Supplementary Table 6: No association was detected between genes containing differentially utilized(DU) exons (A) or any other detected form of alternative splicing(AS)(B) and genes containing differentially methylated regions(DMRs) with respect to the loss of maternal care.

(A)	Genes with DU Exons	Genes without DU exons	(B)	Contains DU Exon or AS Event	Does not contains DU Exon or AS Event
Genes with DMR	8<br>(11.5)	220<br>(216.5)		14<br>(17.2)	214<br>(210.8)
Genes without DMR	251<br>(247.5)	4647<br>(4650.5)		373<br>(369.8)	4527<br>(4530.2)
	Chi-square = 1.1856	p = 0.276224		Chi-square = 0.6765	p = 0.4108

Supplementary Table 7: A strong positive association was detected between genes containing differentially utilized (DU) exons (A) or any other detected form of alternative splicing(AS)(B) and genes that contain greater than 5% methylation in at least one of our six samples.

(A)	Contains DU Exons	No DU Exons	(B)	Contains DU Exon or AS Event	Does not contains DU Exon or AS Event
Methylated Gene	153<br>(113.0)	3568<br>(3608.0)		229<br>(179.2)	3495<br>(3544.8)
Unmethylated Gene	220<br>(260.0)	8337<br>(8297.0)		363<br>(412.8)	8216<br>(8166.2)
	Chi-square = 20.90	p = 4.84e-06		Chi-square = 20.9	p = 4.95e-06

Supplementary Table 8: Spearman correlations for molecular evolution, expression, and methylation. Significance is indicated by asterisks: * indicating $p < 0.05$, ** indicating $p < 0.0001$. Color coding indicates direction and severity of spearman rank correlation. Dark red being a high positive correlation while dark blue indicates a strong negative correlation. dN, dS, and dN/dS were gathered from Rehan et al.[1]. RPKM values were computed using edgeR while mean methylation was global methylation of each gene.

	dN	dS	dN/dS	mean_RPKM	mean_methylation	log2(C_RPKM)	log2(N_RPKM)
dS	0.4103**	1					
dN/dS	0.872**	-0.0168	1				
mean_RPKM	-0.1152**	-0.0437*	-0.1107**	1			
mean_methylation	0.0421*	0.2782**	-0.0789**	0.2392**	1		
log2(C_RPKM)	-0.1094**	-0.0347	-0.1085**	0.9915**	0.2566**	1	
log2(N_RPKM)	-0.1237**	-0.0522*	-0.1155**	0.9931**	0.2314**	0.9719**	1
| log2(C/N) |	0.0636**	-0.0789**	0.1088**	-0.1336**	-0.3881**	-0.1566**	-0.1317**

Supplementary References

1. Rehan, S. M., Glastad, K. M., Lawson, S. P. & Hunt, B. G. The Genome and Methylome of a Subsocial Small Carpenter Bee, *Ceratina calcarata*. *Genome Biol. Evol.* **8,** 1401–1410 (2016).
