# Supplementary material for: The effect of maternal care on gene expression and DNA methylation in a subsocial bee
Source: Nat Commun. 2018 Aug 27;9:3468. doi: 10.1038/s41467-018-05903-0 (PMC6110825; doi:10.1038/s41467-018-05903-0)
Supplement: Supplementary file 3 — Description of Additional Supplementary Files [file 41467_2018_5903_MOESM3_ESM.pdf]

Description of Additional Supplementary Files

File Name: Supplementary Data 1
Description: GO term enrichment analysis compendium. For the differentially expressed genes, alternatively spliced genes (rMATS and DEXseq), differentially utilized exon-containing genes (DEXseq only), differentially methylated region-containing genes, and methylated genes we generated a list of significant genes (listed in the "_Genes" pages) as well as computed GO term enrichment for each gene set using TopGO. GO terms are broken up by category (Cell Component, Biological Process, and Molecular Function) and only terms with a Fisher's exact test P-value < 0.05 are included. The significant genes for each term are also included.

File Name: Supplementary Data 2
Description: Compilation of raw computational results. The Compiled_Analyses page of these data includes the annotation for each gene in the genome (chromosome, start, and end), the RPKM value for that gene in each of the 6 libraries (with MB samples being the maternal-care libraries and PBE samples being those lacking maternal care). Additionally, this page contains the differential expression results for each gene (logFC, logCPM, PValue, and FDR), molecular evolution metrics, genic methylation levels, MethylKit differentially methylated region presence, DEXseq differential exon usage presence, and FlyBase ortholog information for each gene based on a BLAST reciprocal best hit approach. The MethylKit_DMRs page includes the location information and differential methylation analysis results for each region we assayed across the genome. The DEXseq_DUExons page includes the results of differential exon usage analysis across all exons. groupID is the gene name while featureID indicates which exon was analyzed within the given gene. Statistics, counts and relevant transcripts are included in this page as well.
